# Importance of Extraintestinal Manifestations in Early Diagnosis of Gardner Syndrome

**DOI:** 10.1155/2020/7394928

**Published:** 2020-08-04

**Authors:** Bruno Besteiro, Filipa Gomes, Cláudia Costa, Raquel Portugal, Isabel Garrido, Jorge Almeida

**Affiliations:** ^1^Internal Medicine Department, Centro Hospitalar e Universitário de São João, Oporto, Portugal; ^2^Oncology Department, Instituto Português de Oncologia de Coimbra, Coimbra, Portugal; ^3^Pathology Department, Centro Hospitalar e Universitário de São João, Oporto, Portugal; ^4^Gastrenterology Department, Centro Hospitalar e Universitário de São João, Oporto, Portugal

## Abstract

Gardner's syndrome is an autosomal dominant disease caused by a mutation in the *APC* gene with 20–30% of cases presenting de novo. This entity is a variant of familial adenomatous polyposis, with a prevalence of 3/100,000 habitants. It may present as early as 2 months of age with a variety of both colonic and extracolonic symptoms. We report a case of a 21-year-old man, without any known family history, presenting with microcytic hypochromic anemia and constitutional symptoms for two months. Ultimately, after the etiological study, Gardner syndrome diagnosis was established as an index primary familiar case. Gardner syndrome is a clinical challenge which requires a prompt suspicion in order to reach its diagnosis. Given the malignant evolution of adenomas in 100% of untreated patients, early identification of extraintestinal manifestations (identifiable prior to colonic symptoms) is of the essence. A consequent endoscopic study to confirm gastrointestinal involvement is essential for a more favorable prognosis.

## 1. Introduction

Gardner syndrome (GS) represents a phenotypic variant of familial adenomatous polyposis (FAP), caused by mutations of the *APC* tumor suppressor gene, which is inherited in autosomal dominant fashion [1].

In most cases, a family history of GS is present, although 20% to 30% appear to be new mutations of the *APC* gene [2].

It may present at any age with a variety of symptoms, either colonic or extracolonic [1].

The clinical spectrum of disease presentation is variable, and its diagnosis is frequently delayed, despite the presence of clues from early on. Once GS may involve different organs, it is usually quite hard to treat [3].

## 2. Case Report

We report the case of a 21-year-old white male, with a history of excision during childhood of paravertebral neurofibroma, nuchal fibroma and epidermoid cysts, never having met the childhood neurofibromatosis diagnostic criteria. He had no relevant familiar history.

He was admitted in the Internal Medicine Department with a two-month history of constitutional symptoms. The patient also recalled a unique episode of hematochezia which had been attributed to hemorrhoids. He had no other associated symptoms.

On admission, he presented noticeably pale. Physical examination revealed mild hepatomegaly. No other signs were present.

Laboratory test results unveiled microcytic hypochromic anemia (hemoglobin: 8.5 g/dL) with iron deficiency (serum iron < 10 *µ*g/dL and ferritin of 12.3 ng/mL), leukocytosis (14,720/*µ*L), thrombocytosis (6.44 × 10^4^/*µ*L), elevated lactate dehydrogenase (2802 IU/L), mild C-reactive protein elevation (16.7 mg/L), mild hypoalbuminemia (36.6 g/dL), and mild erythrocyte sedimentation rate elevation (34 mm/1 h). Serologic markers for the human immunodeficiency virus and hepatitis B and C virus were negative.

A computed tomography angiography was performed, and it revealed multiple hepatic nodules with neoplastic etiology suspicion and a right adrenal gland nodule.

**Figure 1:**
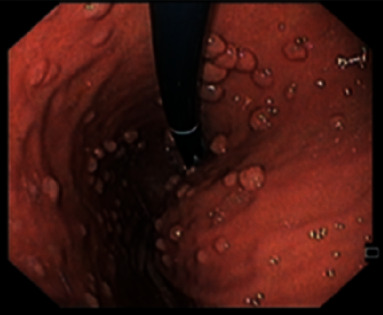
Gastric fundus with nodular aspect and numerous delineated polyps.

**Figure 2:**
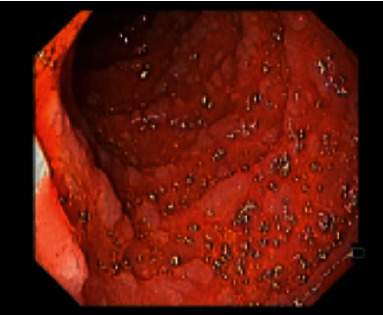
Duodenum with bulbous mucosa and the second duodenal portion with micronodular aspect.

Upper endoscopy displayed numerous delineated polyps, entirely carpeting the stomach and duodenum, consistent with the diagnosis of FAP (Figure 1). Moreover, other abnormal macroscopic findings included the duodenum with bulbous mucosa and the second duodenal portion with micronodular aspect (Figure 2).

**Figure 3:**
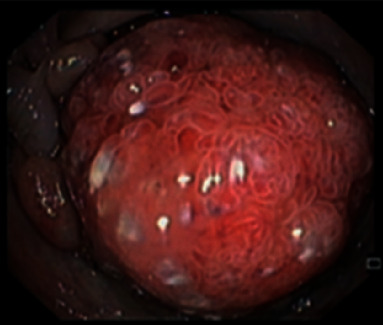
Vegetative lesion of the descending colon.

Lower endoscopy documented nodularity of the terminal ileum, exuberant colic polyposis (>100), and a vegetative lesion of the descending colon (Figure 3).

**Figure 4:**
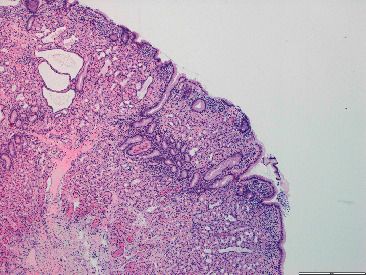
Gastric fundic gland polyp.

**Figure 5:**
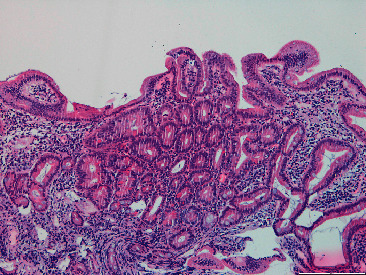
Duodenal mucosa with focal low-grade dysplasia.

Biopsies were taken on both of these exams. Histopathological examination showed fundic glands' polyps (gastric polyps) (Figure 4) and duodenal mucosa with focal low-grade dysplasia (Figure 5). The vegetating colon lesion biopsy revealed adenoma with low-grade dysplasia and a necrosing area in one of the fragments, leading to consideration of a peripheric lesion biopsy.

**Figure 6:**
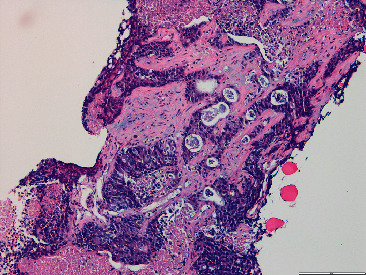
Liver biopsy-adenocarcinoma with necrosis, consistent with gastrointestinal origin.

A liver biopsy was performed resulting in adenocarcinoma of a probable gastrointestinal origin (Figure 6).

The functional study of the adrenal nodule was compatible with the benign adenoma.

The association between intestinal adenomas, gastric fundic gland polyps, adrenal nodule, epidermoid cysts, and fibromas raised the suspicion of GS.

Once the most likely diagnosis was FAP, the patient underwent a thyroid ultrasound due to FAP's association with thyroid carcinoma which did not divulge any abnormality.

The diagnosis was later on confirmed through sequencing of the *APC* gene revealing a pathologic heterozygotic c.4612_4613delGA p.(Glu1538Ilefs^∗^5) variant.

**Figure 7:**
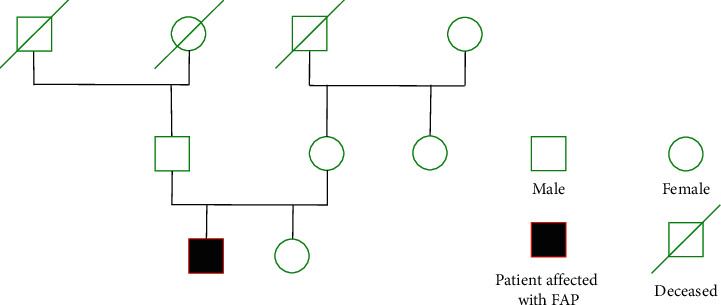
Family pedigree chart.

On discharge, all first-degree family members were referred to oncogenetics in order to be evaluated for FAP (Figure 7). His sister had negative lower endoscopy and genetic testing. His mother did not show any abnormality in colonoscopy, and his father refused to perform the same study. However, the father had a lower endoscopy performed in the previous 5 years without any changes.

At a cancer group meeting, colectomy and chemotherapy were decided with a palliative purpose.

## 3. Discussion

GS is characterized by a constellation of intestinal findings of PAF along with extraintestinal manifestations, including cutaneous lesions and adrenal adenoma, among others [3].

The diagnosis of FAP and GS can be attained through genetic testing for gene mutations or demonstration of multiple colonic polyps. Genetic testing is the most effective method for demonstrating a mutated *APC* gene [1].

This case is significant due to a germline pathogenic mutation c.4612_4613delGA p.(Glu1538Ilefs^∗^5) in the *APC* gene which is absent in population databases (gnomAD, ExAC, and 1000 Genomes), showcasing the allele's low frequency. The gene is altered by a frameshift mutation due to deletion on the 5q22.2 region which potentially originates a nonfunctional truncated protein, therefore being classified as a pathogenic variant.

This case supports that this de novo variation of the *APC* gene should be documented in the literature as it results in a GS phenotype with multiple extracolonic symptoms occurring early in life. Until now, this variation had not been documented as causing this clinical presentation. The location of the mutation within the *APC* gene has been associated with the severity of colonic polyposis, degree of cancer risk, age of cancer onset, survival, and frequency of extracolonic manifestations.

In 1993, an English study which considered APC mutations in FAP families in England had already reported two patients with a severe Gardner's phenotype, both with a deletion in codon 1538 [4].

Although the current data are scarce, we may be facing a more aggressive fast-acting pathologic variant once the average age for cancer diagnosis in individuals with untreated FAP is 39 years. Generally, cancers start to develop a decade after the appearance of the polyps, and if the colon is intact, the majority of patients with FAP will develop colorectal cancer (CRC) by the fifth decade [5].

Hence, dissemination of the information gathered from this case and other similar instances of young patients with FAP and GS will better anticipate further testing and knowledge in order to attain better therapeutic weapons.

In what concerns to extraintestinal symptoms, epidermoid cysts are the most common benign skin lesions in GS [6] and occur most frequently on the face, extremities, and scalp. They show up most often during puberty [7].

In this particular clinical case, the patient presented with paravertebral neurofibroma, nuchal fibroma, and epidermoid cysts as early as an infant which probably then indicated extraintestinal manifestations since this age. Once GS is not typically diagnosed by the presence of these cutaneous lesions, clinicians must be able to recognize the distinct lesions of GS, and they must exclude PAF in a child with fibromas even when the family history is negative since 25% of GS patients can present with a new dominant mutation. Therefore, early detection of these lesions may lead to appropriate further investigations and treatment which might be lifesaving.

Patients with GS are at increased risk of several extracolonic malignancies such as duodenal/periampullary (3–5%), thyroid (2%), pancreatic (2%), hepatoblastoma (1.6%), central nervous system (<1%), gastric (<1%), and adrenal (<1%) [8].

In our patient, all the extracolonic malignancies were excluded.

The thyroid cancer risk is increased approximately 150-fold compared with the general population. Hence, and according to the literature, all patients diagnosed with GS should be screened periodically through physical examination and undergo an ultrasound annually [9].

Adrenal adenomas have been reported in 7% of GS. Despite malignancy of the adrenal incidentaloma is rare in this entity, the functional study should be done once the odds are greater compared with the general population [10].

Though screening guidelines for pediatric population at risk of classic FAP exist, there is no unified approach for any of the extracolonic manifestations. Such methods would be imperative once many early age cases present with these manifestations.

As the syndrome is genetically inherited, diagnosis may also have implications for other family members. Therefore, genetic testing of parents and siblings is a critical part of the evaluation process [1].

This case illustrates a genetic variant of GS rarely reported in the literature and highlights the need for not only a holistic view of sign and symptoms but also an individualized approach to treatment.
